# Comparison of Nanocomposite and Conventional Orthodontic Adhesives: A Prospective Study on Bracket Debonding and Enamel Discoloration

**DOI:** 10.7759/cureus.79537

**Published:** 2025-02-23

**Authors:** Kadegadde H Sudheer, Amesh Golwara, Sovendu Jha, Rashi Chauhan, Richa Shree, Anju Jha

**Affiliations:** 1 Department of Orthodontics and Dentofacial Orthopaedics, Buddha Institute of Dental Sciences and Hospital, Patna, IND; 2 Department of Pedodontics and Preventive Dentistry, Patna Dental College and Hospital, Patna, IND

**Keywords:** amorphous calcium phosphate, debonding, nanocomposites, strereomicroscope, transbond xt

## Abstract

Introduction

The success of orthodontic treatment depends on the adhesive performance of the bonding agents used to bond brackets to enamel. Conventional adhesives, such as Transbond XT (3M Unitek Corp., Monrovia, CA), have been widely utilized owing to their strong mechanical properties and clinical reliability. However, nanocomposites, such as amorphous calcium phosphate (ACP)-modified adhesives, have emerged as promising alternatives, offering potential benefits such as enhanced bond strength, remineralization properties, and better color stability. The present study aimed to compare nanocomposite and conventional adhesives in terms of bracket debonding rates and enamel discoloration over a six-month period.

Materials and methods

This prospective observational study was conducted in an orthodontic department between February and November 2024. Forty patients were included and divided into two groups: group 1 (n = 20 patients), where brackets (N = 400) were bonded using Transbond XT, and group 2 (n = 20 patients), where brackets (N = 400) were bonded using ACP-modified nanocomposite (Aegis Ortho, Bosworth Co. Ltd., Skokie, IL). All patients underwent the same orthodontic bonding protocol and were followed for six months. The bracket debonding time was recorded, and the change in enamel color (ΔE*) was assessed using a spectrophotometer (Vita Easyshade, Vita Zahnfabrik, Bad Säckingen, Germany). The Adhesive Remnant Index (ARI) was evaluated using a stereomicroscope at 20× magnification, and Kaplan-Meier survival analysis was used to assess bracket longevity. Statistical comparisons were conducted at p < 0.05.

Results

The mean debond time was 60.88 ± 20.68 days for Transbond XT and 66.58 ± 34.17 days for the nanocomposite group, with no significant difference (p = 0.876). The nanocomposite group showed lower color change (3.70 ± 0.50) compared to Transbond XT (3.95 ± 0.31), indicating better enamel color stability (p = 0.029). The nanocomposite group had more favorable failure modes, with a higher percentage of adhesive retention on enamel, reducing the risk of microleakage and enamel damage. Kaplan-Meier analysis suggested a slightly longer bracket survival in the nanocomposite group.

Conclusion

Nanocomposite adhesives exhibited bracket retention comparable to that of Transbond XT while demonstrating superior color stability and better ARI scores, indicating improved enamel adhesion and reduced enamel damage upon debonding. Given these advantages, nanocomposites are promising alternatives for orthodontic bonding.

## Introduction

The long-term success of orthodontic treatments relies heavily on the adhesive performance of the bonding agents used to attach brackets to the enamel surface. Traditional adhesive systems, such as Transbond XT (3M Unitek Corp., Monrovia, CA), have been widely used in orthodontics because of their proven clinical efficacy and mechanical reliability [1]. However, with advancements in nanotechnology, nanocomposites have emerged as promising alternatives, offering enhanced mechanical properties, improved bond strength, and potentially superior long-term performance [2]. However, controversial results have been associated with their efficacy compared with conventional composites [2-4]. Furthermore, it is noteworthy that the majority of these investigations are conducted in vitro, which introduces limitations such as a lack of biological variability, exclusion of saliva, absence of physiological forces, inadequate aging simulations, and unrealistic oral environments, thereby diminishing the clinical relevance of the findings.

Unintentional bracket debonding can result from poor oral hygiene, chewing hard or sticky foods, and parafunctional habits like nail-biting. Improper bonding techniques, moisture contamination, and insufficient adhesive application also contribute. Additionally, weak adhesives, high masticatory forces, and improper archwire adjustments can lead to bracket failure, affecting orthodontic treatment efficiency [5,6].

Another problem is microleakage in orthodontic applications, which is characterized by the seepage or discharge of fluids at the interfaces between the tooth, adhesive, and bracket. This can be ascribed to several causative factors, including contraction associated with resin polymerization, differential thermal expansion of enamel and adhesives, and insufficient adhesion [7]. Sudhapalli et al. [8] discovered that, in contrast to conventional adhesives, nanocomposites exhibit a superior peripheral seal to enamel and dentine. Conversely, the investigation conducted by Rajan et al. [9] determined that minimal microleakage was observed with Transbond XT in comparison to nanocomposites.

This microleakage could result in the development of white spot lesions, which are observed not only in the enamel regions adjacent to the bracket but also in the areas located beneath the bracket [10]. This could result in a color change of the enamel in the area of the bracket [11]. Nanocomposites with remineralization, such as bioactive glass (BAG), have been used for orthodontic bonding to address the problem of white spot lesions. However, in a systematic review by Alamri et al. [3], it was concluded that while empirical research has substantiated the efficacy of BAG orthodontic bonding resins in promoting enamel remineralization, a notable degree of variability was observed among studies owing to the absence of a standardized protocol for in vitro investigations.

However, owing to the lack of comprehensive research evaluating the extent of enamel discoloration after six months of orthodontic treatment when bonded with nanocomposites versus conventional adhesives, this study was conducted to compare the adhesive performance of nanocomposites and Transbond XT in terms of bracket debonding rates and enamel discoloration after six months of orthodontic treatment. By analyzing these factors, this study aimed to provide valuable clinical insights into the feasibility of incorporating nanohybrid adhesives into routine orthodontic practice.

## Materials and methods

Study design and setting

This prospective observational study was conducted in the Department of Orthodontics at Buddha Institute of Dental Sciences and Hospital, Patna, from February 2024 to November 2024. The study obtained ethical approval from the institutional ethics committee and followed the principles of the Declaration of Helsinki. Written informed consent was obtained from all patients.

Sample size estimation

Sample size estimation was conducted using G*Power software version 3.1 (Heinrich Heine Universität Düsseldorf, Düsseldorf, Germany) to achieve a statistical power of 80%, with a significance level (alpha error) of 5%. Based on a minimum effect size of 0.63, derived from a prior study by Niknam et al. [12], a total sample size of 18 patients per group was determined to be adequate. The referenced study evaluated the value of ΔE (enamel color change) of Transbond XT and nanocomposites and reported a mean difference of 2.95 (14.96-12.01) in ΔE with a pooled standard deviation of 4.6. Considering the 10% loss to follow-up, 20 patients per group were included in this study.

Eligibility criteria

Patients aged 12-25 years requiring fixed non-extraction orthodontic treatment with minor crowding, gingival, and plaque index of less than one were included in this study. Patients with prior fixed orthodontic treatment, enamel hypoplasia, bruxers, fluorosis, prior enamel discoloration, poor oral hygiene, history of smoking, systemic conditions affecting enamel, crowns on teeth, and heavily restored teeth were excluded from the study.

Methodology

All patients underwent thorough oral prophylaxis before starting orthodontic treatment. All patients were started on 0.022 × 0.028-inch Roth mechanotherapy (3M Unitek Corp., Monrovia, CA) with a non-extraction approach. All patients were started by an independent orthodontist who routinely performed orthodontic bonding procedures in the department with more than five years of experience. The investigator had no role in treatment selection or bonding procedures and only observed and recorded outcomes. The sequential allocation method was used where the first 20 patients who required orthodontic treatment were bonded with conventional adhesive as group 1, and the next 20 patients who required orthodontic treatment were bonded with the ACP nanocomposite as group 2. In group 1 (n = 20 patients), all brackets (N = 400) were bonded with Transbond XT, and in group 2 (n = 20 patients), all brackets (N = 400) were bonded with an amorphous calcium phosphate (ACP)-modified orthodontic nanocomposite (Aegis Ortho, Bosworth Co. Ltd., Skokie, IL).

In group 1, the bonding procedure started with thorough enamel cleaning using pumice and water, followed by etching with 37% phosphoric acid for 30 seconds to create micro-retentive porosities. The enamel was then rinsed and dried until it appeared frost-white. A thin layer Transbond XT Primer (3M Unitek Corp., Monrovia, CA) was applied and gently air-dried to enhance the adhesion. Next, the Transbond XT adhesive paste was placed on the bracket base, which was then positioned on the tooth surface. The bracket was firmly pressed into place, and the excess adhesive was removed. Finally, light curing was performed for 10 seconds per side of the bracket (mesial and distal) using a light-emitting diode (LED) curing light (1200 mW/cm²) (3M™ Elipar™ DeepCure-S, 3M Oral Care, St. Paul, MN) to achieve complete polymerization and bond strength.

The bonding procedure was the same for group 2; however, the nanocomposite did not require a separate primer as the ACP-containing formulation enhanced bonding while actively releasing calcium and phosphate ions for enamel protection. The adhesive paste was applied directly to the bracket base and positioned on the etched enamel. Gentle pressure was applied to ensure proper adaptation, and excess material was removed before light-curing for 10 seconds per side of the bracket. After orthodontic bonding, all patients underwent an alignment phase where 0.014-inch nickel titanium (NiTi) was placed for two months, followed by 0.016 × 0.022-inch NiTi for two months and 0.016-inch stainless steel wires for another two months. All patients were given similar post-bonding instructions, such as avoiding chewing on hard and sticky food, chewing on both sides, and maintaining proper oral hygiene, which included brushing twice with a soft-bristled toothbrush and warm saline rinses. The patients were recalled every four weeks.

**Figure 1:**
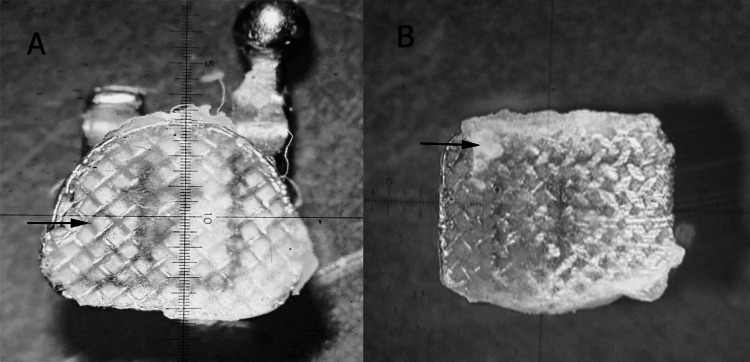
Debonded brackets analyzed for Adhesive Remnant Index (ARI). (A) Score 0 in bracket from the nanocomposite group. (B) Score 1 in bracket from the Transbond XT group. Stereomicroscopic image under 20× magnification. This figure presents a bracket that was debonded from a patient in the study.

All patients were followed up for six months to check for any unintentional bracket detachment, along with the cause if identifiable. The debonded bracket bases were examined under a stereomicroscope at 20× magnification (Biotron Healthcare, Mumbai, India) for Adhesive Remnant Index (ARI) scoring (Figure 1), where score 0 was given when no adhesive was seen on the bracket base, score 1 was given when less than 50% was present on the bracket base, score 2 was given when more than 50% adhesive was present on the bracket base, and score 3 was given when all the adhesive was seen on the bracket base [13].

The bracket/adhesive interface may be regarded as the most advantageous site for secure debonding, resulting in the retention of the majority of the adhesive on the enamel substrate while minimizing the residue on the bracket base, as evidenced by scores 0 and 1. This type of debonding reduces the likelihood of enamel fractures [14]. The baseline chromaticity of the buccal/labial surface of all teeth was evaluated employing a spectrophotometer (Vita Easyshade, Vita Zahnfabrik, Bad Säckingen, Germany) against a standard white ceramic background (L* = 89, a* = −2.6, b* = 5), wherein L* denotes the brightness, a* signifies the red-green spectrum, and b* represents the yellow-blue color spectrum. The residual adhesive was meticulously eliminated from the dental surfaces using a 12-blade tungsten carbide bur and subsequently polished, specifically at the sites of bracket failure. The chromatic attributes of the buccal surfaces were re-evaluated using a spectrophotometer. All teeth were evaluated at the same site in the middle third of the facial surface of the teeth, and measurements were taken under the same lighting conditions and in the same room for standardization. The change in color (∆E*) was determined using the numerical values of L*, a*, and b* for each specimen. It was calculated using the formula ΔE = square root of (L1−L2)2 + (a1−a2)2 + (b1−b2)2, where L1, a1, and b1 represent the initial color values recorded before bonding, and L2, a2, and b2 correspond to the values measured after debonding of bracket. Each measurement was conducted three times, and the average value was used for analysis to ensure accuracy and consistency in the color assessment. The time of bracket debonding is also noted. The clinical color match was assessed based on ΔE* values, which indicate the degree of color difference. A ΔE* value of zero to one was considered excellent, ensuring near-perfect color consistency. Values between one and two were classified as good, indicating a minimal but acceptable variation. A ΔE* range of two to 3.5 was deemed clinically acceptable, as the color difference remained within tolerable limits. However, any ΔE* exceeding 3.5 was regarded as a noticeable mismatch, indicating a perceptible color discrepancy that may not meet aesthetic expectations [15].

Statistical analysis

**Figure 2:**
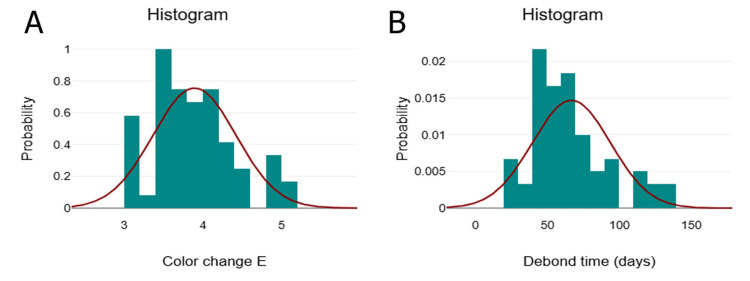
Non-normal distribution of data by Kolmogorov-Smirnov test. (A) Color change of enamel surface. (B) Debond time of bracket in days. This figure is derived from the study data.

Descriptive statistics, including 95% confidence intervals (CIs), were calculated for various study parameters. Data normality was assessed using the Kolmogorov-Smirnov test, and histogram evaluation confirmed a non-normal distribution (Figure 2). The Mann-Whitney U test was used to compare groups in terms of debonding time and ΔE* values, while chi-square analysis was performed to evaluate the frequency distribution of the ARI score. Additionally, Kaplan-Meier survival analysis was conducted to assess the bracket survival time across different groups. Statistical analysis was performed using the SPSS software (IBM SPSS Statistics for Windows, IBM Corp., Version 25, Armonk, NY), with the level of significance set at p < 0.05.

## Results

The mean debond time was 60.88 ± 20.68 days for the Transbond XT group and 66.58 ± 34.17 days for the nanocomposite group. The color change (ΔE*) showed a mean value of 3.95 ± 0.31 in the Transbond XT group, while the nanocomposite group exhibited a mean ΔE* of 3.70 ± 0.50. The nanocomposite group demonstrated a slightly longer mean debonding time compared to the Transbond XT group, although with greater variability. In terms of color stability, the Transbond XT group exhibited a slightly higher mean ΔE* value, indicating a greater color change than the nanocomposite group (Table 1).

**Table 1:** Descriptive analysis of study groups for outcome variables Data are represented in the form of mean and standard deviation (SD). N': total number of debonded brackets in each group, N: total number of bonded brackets in each group

Variables	Groups	N'/N	Mean	SD	Minimum	Maximum	95% CI for mean
Debond time in days	Transbond XT	32/400	60.88	20.68	24.00	98.00	53.42-68.33
	Nanocomposite	26/400	66.58	34.17	23.00	134.00	52.77-80.38
Color change ΔE*	Transbond XT	32/400	3.95	0.31	3.45	4.97	3.84-4.06
	Nanocomposite	26/400	3.70	0.50	3.12	4.89	3.49-3.90

The mean rank for debonding time was 29.90 in the nanocomposite group and 29.17 in the Transbond XT group, with a p-value of 0.876, indicating no statistically significant difference between the two groups. The nanocomposite group had a mean color change (ΔE*) of 24.08, while the Transbond XT group had a mean rank of 33.91, with a p-value of 0.029, suggesting a statistically significant difference. There was no significant difference in debonding time between the two adhesive groups. However, color change (ΔE*) was significantly higher in the Transbond XT group than in the nanocomposite group, indicating that nanocomposite exhibited better color stability (Table 2).

**Table 2:** Comparison of study groups with the Mann-Whitney U test *p-value < 0.05: significant.

Variables	Groups	Mean rank	Sum of rank	z-value	p-value	r-value
Debond time in days	Transbond XT	29.17	933.5	-0.16	0.876	0.02
	Nanocomposite	29.90	777.5			
Color change ΔE*	Transbond XT	33.91	1085.0	-2.21	0.029*	0.29
	Nanocomposite	24.08	626.0			

In the Transbond XT group, 11 (18.97%) brackets had ARI scores of zero, and 15 (25.86%) brackets had an ARI score of 1, indicating minimal adhesive retention on the bracket surface and good adhesion with enamel, which is the desired mode of debonding. In contrast, the nanocomposite group had a higher percentage of brackets with ARI scores of two in nine (15.52%) brackets and three in four (6.9%) brackets, suggesting less adhesive retention with enamel. The chi-square statistic was 7.52, with a p-value of 0.057, indicating no significant difference between the groups. This suggests that the Transbond XT showed good adhesion with the enamel and a better mode of failure (Table 3).

**Table 3:** Comparison of ARI score with chi-square analysis Data are represented in the form of N' (%). p-value > 0.05: non-significant. ARI: Adhesive Remnant Index, N': total number of debonded brackets in each group

Groups	ARI score								Chi-square statistic	p-value
	0		1		2		3			
	N'	%	N'	%	N'	%	N'	%		
Nanocomposite	8	13.79	5	8.62	9	15.52	4	6.90	7.52	0.057
Transbond XT	11	18.97	15	25.86	4	6.90	2	3.45		

**Figure 3:**
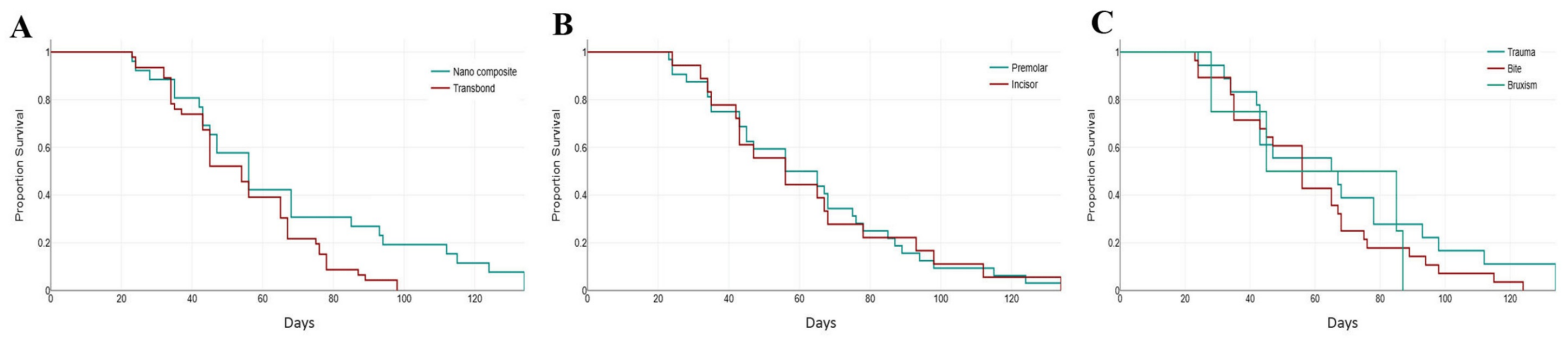
Kaplan-Meier survival analysis revealed variations in bracket survival based on the (A) adhesive type, (B) tooth type, and (C) cause of debonding. This figure is derived from the study data.

Kaplan-Meier survival analysis revealed variations in bracket survival based on the adhesive type, tooth type, and cause of debonding. In the group comparison, the nanocomposite group demonstrated a slightly longer bracket survival than the Transbond XT group, indicating better adhesive performance over time. When comparing tooth types, incisors showed a slightly longer survival than premolars, although the difference was minimal. Regarding the debonding causes, trauma resulted in the most rapid bracket loss, followed by biting forces. These findings suggest that bracket survival is influenced by the choice of adhesive, type of tooth, and cause of debonding, with nanocomposite adhesives, incisors, and lower impact forces contributing to longer bracket retention (Figure 3).

## Discussion

The visual appeal of teeth following the removal of orthodontic appliances and the retention of orthodontic brackets throughout the therapeutic process constitutes a significant concern for both the patient and orthodontic practitioner. The recurrent detachment of brackets may result in prolonged treatment duration and alterations in coloration attributable to the demineralization of enamel, subsequently leading to the formation of white spot lesions that adversely affect aesthetic outcomes [11,16]. This study aimed to compare the adhesive performance of nanocomposite-based orthodontic adhesives with the conventional Transbond XT in terms of bracket debonding rates and enamel discoloration over a six-month period. These results provide valuable insights into the clinical feasibility of incorporating nanocomposite adhesives into routine orthodontic practice.

The primary concern when evaluating orthodontic bonding systems is their ability to maintain bracket retention over the course of the treatment. In this study, the mean debonding time was slightly longer in the nanocomposite group than in the Transbond XT group. Nanocomposite adhesives, such as ACP-modified formulations, have been designed to offer mechanical strength comparable to conventional adhesives while also releasing bioactive ions that may contribute to enamel protection [17]. Although previous studies have reported lower bond strengths of nanocomposites compared to traditional composites [11,18,19], the findings of this study indicate that nanocomposites provide an acceptable level of adhesive performance. The slight variability in debonding times within the nanocomposite group may be attributed to individual differences in oral habits, occlusal forces, and patient compliance. This disparity in findings might be due to the fact that the nanocomposite used in our study contained silica nanoparticles, imparting mechanical strength. A research investigation conducted by Marovic et al. [20] determined that the enhancement of load-bearing characteristics necessitated the integration of a more robust barium-glass filler within a composite that included ACP, resulting in an augmentation of both flexural strength and elastic modulus while concurrently exhibiting no detrimental effect on ion release profiles.

Microleakage remains a concern in orthodontic bonding as it can lead to bracket failure and the development of white spot lesions [7]. The ARI scores provide insight into the mode of bracket failure and the extent of adhesive retention on the enamel. Ideally, the adhesive should remain on the enamel rather than on the bracket to minimize the risk of enamel damage upon debonding [14]. In this study, the ARI scores revealed that the nanocomposites exhibited a less favorable failure mode than Transbond XT. This suggests that nanocomposites promote lower adhesion with enamel, which is advantageous for preventing premature debonding. Conversely, the Nanocomposite group had higher proportions of ARI scores of two and three, indicating that more adhesive remained on the bracket base than on the enamel. This type of failure mode is less desirable as it suggests weaker adhesion to the enamel surface, increasing the likelihood of microleakage and subsequent enamel demineralization. In addition, it can lead to enamel tears and fractures [14]. Conversely, Rajan et al. [9] reported less microleakage with conventional brackets than with nanocomposites. The disparity in the results might be due to the use of a different nanocomposite in their study, and it was an in vitro study.

One of the major concerns in orthodontic treatment is enamel discoloration after bracket removal. In this study, enamel color change was assessed using spectrophotometric analysis, which provides an objective measure of discoloration. The results revealed a statistically significant difference in ΔE* values between the two groups, with the Transbond XT group exhibiting greater color change than the nanocomposite group. Enamel discoloration following orthodontic bonding can be attributed to several factors, including adhesive degradation, plaque accumulation, and penetration of pigmented substances into microgaps at the adhesive-enamel interface [21]. This can lead to enamel demineralization and the development of white spot lesions [12], which can lead to color changes in the enamel. The slightly higher ΔE* values observed in the Transbond XT group may be due to its resin composition, which can undergo polymer degradation and subsequent color alteration over time [22]. Additionally, microleakage and adhesive retention patterns, as indicated by ARI scores, may have influenced the extent of enamel discoloration. Because nanocomposites exhibited better marginal sealing and adhesive retention on the enamel, they likely reduced the penetration of staining agents, thereby enhancing color stability.

Moreover, the remineralization potential of ACP could have led to a decreased color change of the enamel. ACP has demonstrated anti-cariogenic properties along with its potential for remineralization [23]. Bioactive materials containing ACP facilitate mineralization by augmenting the concentrations of calcium and phosphate within the lesion, particularly in the acidic milieu of the oral cavity, to levels that surpass those present in the surrounding oral fluids, thereby altering the thermodynamic forces of the solution to favor apatite formation. Furthermore, ACP can maintain these supersaturation conditions for prolonged durations [24]. Kaplan-Meier survival analysis was used to evaluate bracket survival over the six-month period. The results indicated that the nanocomposite group exhibited slightly longer bracket survival than the Transbond XT group. Although this difference was not statistically significant, it suggests that the nanocomposites may offer marginally improved long-term retention.

Strengths of the study

The merits of this study are its prospective in vivo methodology, uniform bonding protocols, and objective evaluation techniques, including spectrophotometry for assessing color stability and Kaplan-Meier survival analysis for evaluating bracket durability. 

Clinical implications of the study

The comparable debonding rates between the nanocomposites and Transbond XT suggest that nanocomposites can be a viable alternative to conventional adhesives. Given the minimal alteration in color observed in enamel associated with nanocomposites, these materials present a promising alternative for use as adhesives in orthodontic bonding, particularly considering that the duration of orthodontic treatment typically spans one to two years. Furthermore, the improved ARI scores observed in the nanocomposite group indicated a reduced risk of enamel damage upon debonding, which is a crucial consideration for patient safety.

Limitations and future recommendations

Despite these advantages, this study has some limitations. The study was conducted over a six-month period, and the long-term adhesive performance beyond this timeframe remains unclear. Additionally, although in vivo conditions were simulated as closely as possible, factors such as dietary habits, oral hygiene, and patient compliance may introduce variability in clinical outcomes.

Future investigations should prioritize the increase in sample size and implementation of long-term follow-up intervals to evaluate the longevity of nanocomposite adhesives throughout orthodontic treatment. Furthermore, the standardization of in vitro testing methodologies for nanocomposite adhesives, such as the assessment of bond strength, remineralization potential, and microleakage, will help provide more information.

## Conclusions

This investigation demonstrated that nanocomposite adhesives offer bracket retention comparable to that of conventional Transbond XT while also exhibiting enhanced color stability and a more advantageous mode of failure. Despite the absence of a statistically significant disparity in debonding rates, nanocomposites revealed marginal superiority in terms of bracket survival and enamel adhesion. Based on these results, nanocomposites have emerged as promising alternatives for orthodontic bonding applications.
